# Phytochemistry, Ethnopharmacology, and Pharmacology of *Lessertia frutescens* (Cancer Bush): A Comprehensive Review

**DOI:** 10.3390/plants14142086

**Published:** 2025-07-08

**Authors:** Kadidiatou O. Ndjoubi, Rajan Sharma, Ahmed A. Hussein

**Affiliations:** Chemistry Department, Cape Peninsula University of Technology, Bellville Campus, Symphony Road, Bellville 7535, South Africa; dickakadi@yahoo.fr (K.O.N.); sharmar@cput.ac.za (R.S.)

**Keywords:** *Lessertia frutescens*, *Sutherlandia frutescens*, cancer bush, ethnopharmacology, phytochemistry, phytotherapy

## Abstract

*Lessertia frutescens* (L.) Goldblatt & J.C.Manning (synonym *Sutherlandia frutescens*), commonly known as cancer bush, is one of the most prominently used South African medicinal plants, with a rich history of traditional uses among indigenous communities. Its phytochemical profile showed different metabolites such as amino acids, fatty acids, sugars, flavonoid glycosides, cycloartenol glycosides, and oleanane-type saponins. Moreover, several research studies have highlighted the promising therapeutic effects of *L. frutescens* in combating various cancer cell lines. Additionally, the plant demonstrated potent immunomodulatory, antioxidant, anti-inflammatory, antidiabetic, neuroprotective, antistress, and antimicrobial activities. These research findings highlight *L. frutescens* as a promising candidate for the development of new or complementary therapies for a range of diseases and conditions. This review analyses the chemical and biological properties of *L. frutescens* based on 154 articles identified through SciFinder. Of these, 78 articles, including two patents, met the inclusion criteria and were reviewed. Studies focused on agriculture and horticulture were excluded as they fell outside the scope of this research.

## 1. Introduction

*Lessertia frutescens* (formerly known as *Sutherlandia frutescens*)*,* an indigenous Southern African medicinal plant belonging to the Fabaceae family, is prominent in traditional medicine [1]. Commonly referred to as “cancer bush”, it ranks among the foremost medicinal plants in South African herbal pharmacopoeia [2]. It is widely distributed across the Eastern Cape, KwaZulu-Natal, Northern Cape, and Western Cape, where it plays a crucial role in ameliorating the body’s ability to combat diseases and ailments. Moreover, it aids in reducing mental and physical stress by helping the body to mobilise its physiological and immunological resources [1,3,4].

Despite its extremely bitter taste, the leaves and stems of *L. frutescens* have been extensively studied for their therapeutic properties. Traditionally, prepared as a medicinal tea, the plant has been used for treating internal cancers and as a cancer prophylactic [2]. It is well known as an adaptogenic tonic, with commercial tablets frequently used to counteract the muscle-wasting associated with HIV-AIDS and to stimulate appetite. It is considered safe for consumption, with only mild side effects such as sporadic dry mouth, dizziness, mild diuresis, and diarrhoea in cachectic patients [3,5]. Furthermore, the plant has been used as a drug support in the treatment of anorexia, cancer, influenza, HIV/AIDS, and tuberculosis [6].

Scientific validation of these traditional claims has been pursued through various studies. In 2002, several clinical trials were conducted to verify the assertions made by indigenous people regarding the safety, potency, and therapeutic uses of *L. frutescens*. Following a three-month clinical trial assessing the plant’s toxicity using vervet monkeys, it was discovered that ingestion of the plant extract at human-equivalent dosages showed no toxicity or adverse effects. Consequently, the South African Medical Research Council (MRC) affirmed the safety of *L. frutescens* decoctions, infusions, and tinctures for consumption [7,8].

In vitro and in vivo studies have further highlighted its therapeutic potential, particularly in mitigating metabolic and oxidative stress-related disorders. Moreover, the isolated bioactive constituents such as mucronulatol, D-pinitol, sutherlandioside B, α-linolenic acid, *L*-canavanine, and GABA have been identified as key contributors to its neuroprotective, antidiabetic, antistress, anti-TB, and anticancer activities [4,9,10,11,12,13].

Despite its widespread use and documented therapeutic benefits, critical gaps remain in the literature. Previous reviews have predominantly focused on the plant extract’s anticancer, anti-inflammatory, anti-HIV, and immunomodulatory properties, often overlooking challenges like the high therapeutic concentrations required for efficacy in certain treatments. Furthermore, the documentation on *L. frutescens* secondary metabolites and their pharmacological properties is incomplete, emphasising the need for a comprehensive evaluation.

This review seeks to bridge existing knowledge gaps by critically synthesising the available literature on *Lessertia frutescens*, with a focus on its phytochemical composition, ethnobotanical significance, and pharmacological properties. Specifically, it aims to document the plant’s phytochemical constituents, assess the experimental approaches employed in studying its biological activities, including details such as concentrations, cell lines, organisms, and techniques, and to highlight its pharmacological potential while identifying limitations and gaps in current research.

## 2. Methodology

### 2.1. Search Strategy and Data Selection

This review was conducted without restrictions on geographical scope or time frame, with the search for articles concluding in December 2024. A total of 154 articles on *S. frutescens* (130) and *L. frutescens* (24) were identified using SciFinder. Of these, 78 articles, including 2 patents, were selected based on predefined inclusion criteria. These criteria focused on studies related to the phytochemistry, ethnopharmacology, and pharmacology of the plant, irrespective of the experimental methods, tested concentrations, or types of extracts.

Articles primarily addressing agriculture, horticulture, or other unrelated fields (66 in total) were excluded as they fell outside the scope of this review. Each selected article underwent a meticulous evaluation to ensure relevance and quality. References cited in the primary sources were further analysed to identify additional studies that aligned with the inclusion criteria.

### 2.2. Data Extraction

The data extraction process was designed to collect and organise detailed and relevant information aligned with the review’s objectives. Key data points included the phytochemical composition of the plant, as well as the therapeutic potentials of its extracts, fractions, and isolated compounds. Experimental approaches, including in vitro, in vivo, and clinical trials, were carefully documented. Data from these studies were categorised and tabulated to distinguish findings from in vitro, in vivo, and clinical trials, respectively.

Treatment protocols were meticulously detailed, encompassing doses or concentrations, treatment duration, targeted pharmacological activities, and the techniques used to evaluate these activities. Specific data points for the in vitro studies included the cell lines used, while the in vivo studies recorded the animal models and the conditions to which they were subjected. For clinical studies, data were gathered on the number of patients involved and their health status, specifying whether they were healthy or affected by a particular disease. Mechanisms of action for bioactive compounds and extracts were noted wherever reported. All the extracted data were carefully tabulated to ensure consistency and avoid duplication.

## 3. Taxonomy, Nomenclature, and Distribution

The *Lessertia* genus, belonging to the Fabaceae family, comprises 62 accepted species [14]. Some of these species were formerly classified under the *Sutherlandia* genus, such as *Lessertia frutescens*, previously known as *Sutherlandia frutescens* [14,15]. Prior to the reclassification of the *Sutherlandia* species, 35 species within the *Lessertia* genus were endemic to South Africa [16].

*L. frutescens* stands out as one of the most extensively studied and utilised medicinal plants in South Africa. It is known by names such as umnwele in Xhosa, blaasbossie in Afrikaans, Insiswa in Zulu, and Musa-Pelo in Sotho [17]. It is also associated with around twenty-five common names in languages like Afrikaans, Zulu, Tswana, and Sotho, which often reflect aspects of its characteristics, including its seedpods (blaasbossie and blaas-ertjie), flower colour or shape (kalkoenbos, hoenderbelletjie, and eendjie), appearance (unwele), taste (bitterbos), or medical uses (kankerbos, insiswa, phetola, and lerumo lamadi) [18].

## 4. Botanical Description

**Figure 1:**
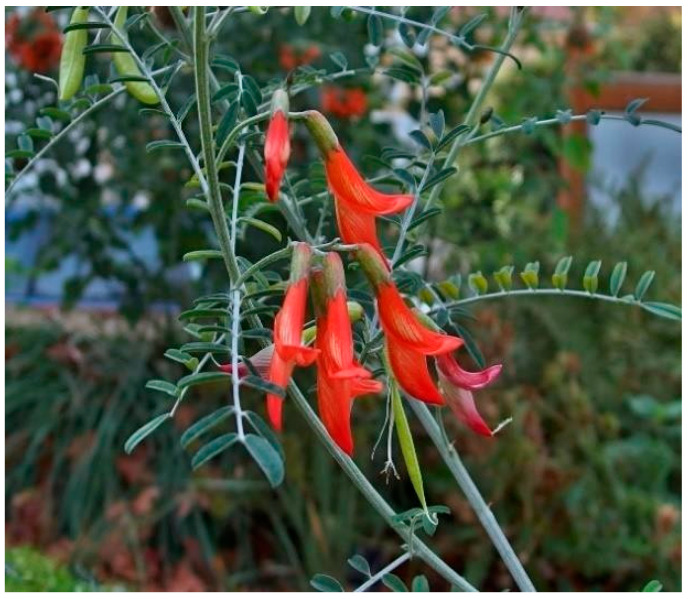
Lessertia frutescens [23].

*L. frutescens* (Figure 1) is a perennial non-climbing shrub, typically reaching heights between 0.2 and 2.5 m [19]. Its leaves are greyish green, pinnately compound, with each leaflet measuring 4–10 mm. These leaflets vary from elliptic to narrowly oblong or ovate oblong, with the adaxial leaflets’ surface ranging from glabrous to sericeous depending on the plant’s cultivation region [4]. The prostate to erect stems is either sparsely pubescent or glabrous with many leaves in terminal racemes [4,20]. Its orange-red butterfly-shaped flowers (35 mm long) appear in short clusters within the leaf axils at branch tips from September to December [21]. After flowering, the plant produces inflated bladder-like pod fruits that contain black seeds [19,22].

## 5. Ethnomedicinal Uses

**Figure 2:**
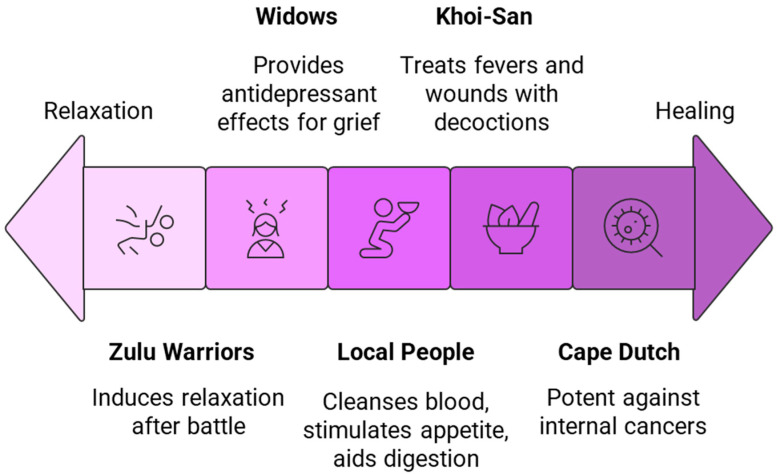
Traditional uses of *L. frutescens* range from relaxation to healing.

In South Africa, *L. frutescens* has been a staple in traditional medicine, utilised by healers, herbalists, diviners, and local people to treat various ailments and diseases. Despite its bitterness, it has gained popularity as a medicinal tea due to its liquorice aftertaste [15]. Since 1895, the Khoisan and Cape Dutch have known this flowering shrub as a cancer bush due to its potency against internal cancers [4,24,25]. Conversely, the Nama and Khoi-San communities traditionally use decoctions of the plant to treat fevers and wounds [25]. Historically, Zulu warriors would consume a concoction of the plant to induce relaxation following battle. In contrast, the widows of the deceased warriors used it as an antidepressant to help them navigate through their grief. In Van Wyk and Albrecht’s [4] review on the ethnobotany of *L. frutescens*, it was revealed that decoctions or infusions of the leaves were used in the treatment of diarrhoea, urinary tract infection, rheumatism, inflammation, intestinal pain, haemorrhoids, eye diseases, chickenpox, and skin disorders (Figure 2). Moreover, decoctions of *L. frutescens* have been used to treat various diseases and ailments such as asthma, chronic bronchitis, colds, coughs, convulsion, diabetes mellitus, epilepsy, gastric, gout, heart failure, heartburn, hypertension, kidney and liver infections, menopausal symptoms, osteoarthritis, pains, peptic ulceration, rheumatoid arthritis, reflux oesophagitis, varicose veins, and stress-related conditions linked to the endocrine system [6,8,26,27]. The plant serves as a tonic that cleanses the blood, stimulates appetite, and aids digestion [4,18,28].

## 6. Phytochemistry

**Figure 3:**
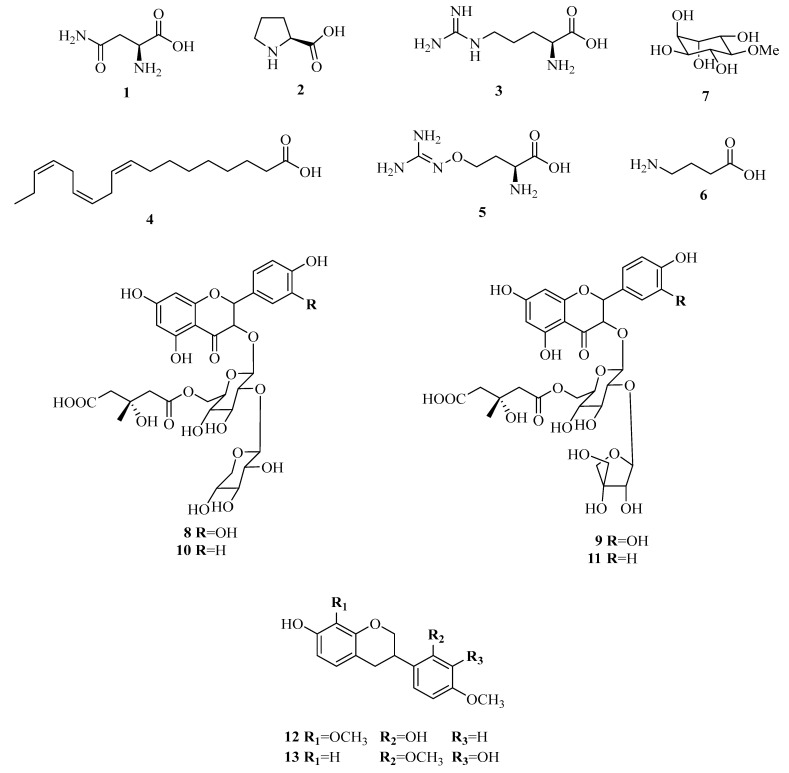
Chemical structures of *L. frutescens* isolated compounds (**1**–**13**). Created using ChemOffice Pro 2015 (version 15.00).

The phytochemical studies conducted on *L. frutescens* have revealed that the leaves contain a high concentration of free amino acids such as *L*-asparagine (1.6–35 mg/g), (**1**); proline (0.7–7.5 mg/g), (**2**); and *L*-arginine (0.5–6.7 mg/g), (**3**) [4]. Additionally, the essential omega-3 fatty acid α-linolenic acid (**4**) was isolated from the dichloromethane–methanol (1:1) extract of the aerial part of the plant [11]. For the first time, Moshe [29] isolated *L*-canavanine (**5**), a non-protein amino acid usually found in the seed, from the leaves of *L. frutescens*. Furthermore, *γ*-aminobutyric acid (GABA), (**6**) was identified as another non-protein-free amino acid in the leaves. The cyclitol D-pinitol (**7**), known for its antidiabetic activity, was also isolated from the plant leaves. In addition to these compounds, researchers Fu et al. [30] isolated and identified four flavonoid glycosides, named sutherlandins A-D (**8**–**11**) (Figure 3).

**Figure 4:**
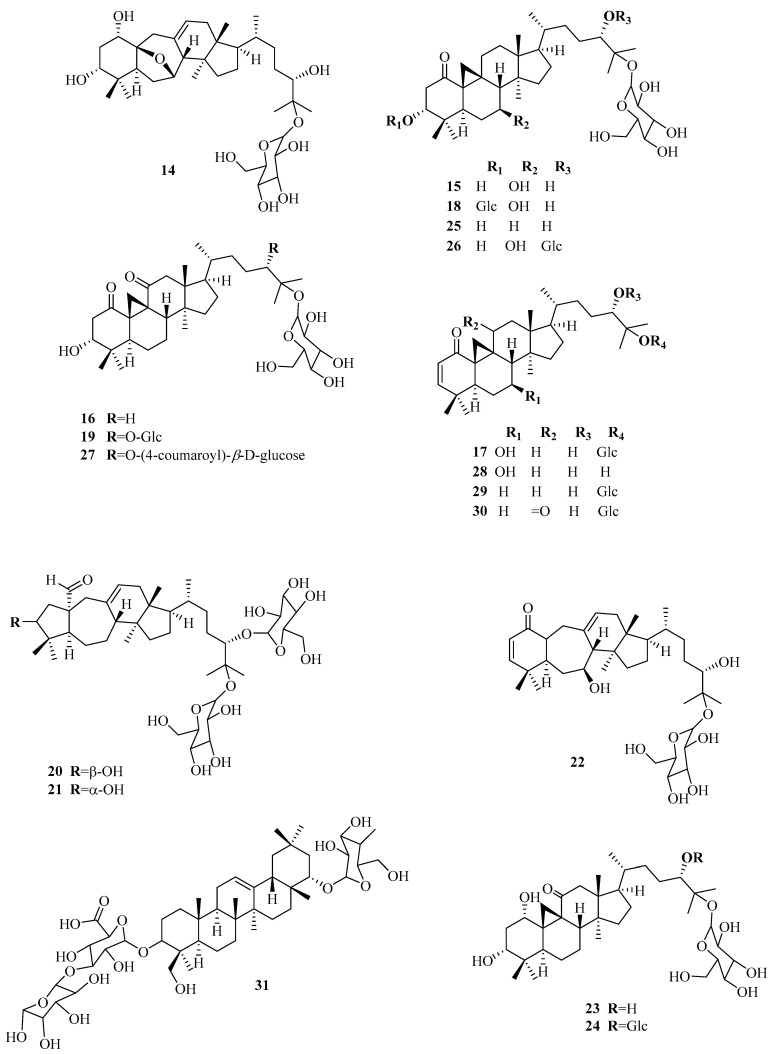
Chemical constituents of *L. frutescens* (**14**–**31**). Created using ChemOffice Pro 2015 (version 15.00).

Fu et al. [31,32,33] reported the isolation of 8 cycloartane glycosides given the trivial name of sutherlandiosides A-H (**14**–**21**) (Figure 4). Recently, the oleanan-type saponin 3-*O*-[α-*L*-rhamnopyranosyl-(1-3)-*β*-D-glucurono pyranosyl]-22-*epi*-soyasapogenol B-22-*O*-*β*-D-glucopyranoside (**31**), along with seven cycloartane glycoside compounds named sutherlandiosides E-K (**22**–**27**) were isolated [34]. However, some of these trivial names, specifically sutherlandiosides E-H (**22**–**25**), have already been assigned by Fu [33] to another four cycloartane triterpenoid diglycosides (**18**–**21**). Moreover, the cycloartane glycoside sutherlandioside I reported as new by Tchegnitegni et al. [34] had already been identified as sutherlandioside G (**18**) by Fu et al. [32]. The IUPAC names of the sutherlandiosides E-H isolated by Fu [33] and Tchegnitegni et al. [34] are tabulated in Table 1 to highlight the duplication in sutherlandiosides naming. Recently, Ndjoubi et al. [13] isolated two cycloartane glycosides, namely lessertiosides A (**29**) and B (**30**), as well as the flavonoids 8-methoxyvestitol (**12**) and mucronulatol (**13**).

In 2019, Gonyela et al. [35] reported the isolation of cycloartenol (**28**) from *L. frustecens* leaves. However, D-pinitol, *L*-canavanine, and sutherlandioside B are identified as the major components in the plant [36]. Apart from these secondary metabolites, *L. frutescens* is known to biosynthesise tannins [9,37]. As mentioned above, the chemistry of triterpenoids of this plant is unique, especially the oxygenation pattern of rings A and C. Compounds **20** and **21** have unique rearrangements in their cycloartenol glycoside structures, featuring a rearranged five- and seven-membered A/B-ring system. This discovery marked the first observation of the hexadecahydro-1-*H*-indeno [5,4-f] azulene ring system in nature [32]. Interestingly, compounds **16**, **19,** and **27** have a unique oxygenation pattern in rings A and C with two carbonyls at C-1 and C-11, and 3α-OH, while compounds **23** and **24** have 1α, 3α-diOH in addition to C=O in C-11. Another feature of the isolated triterpenoids is the configuration of the 3-OH, which is assigned to the uncommon α-position in most of the isolated compounds, except **20**, which may need further revision.

## 7. Ethnopharmacological and Pharmacological Properties of *L. frutescens* Extracts and Compounds

*L. frutescens*, a medicinal plant native to Southern Africa, boasts diverse ethnomedicinal applications. In South Africa, the Khoisan and Cape Dutch people have historically used this perennial shrub for treating internal cancers, wounds, inflammation, stomach pains, diabetes, HIV/AIDS, and infections [4]. While the pharmacology and ethnomedicinal properties of *L. frutescens* (Table 2 and Table 3) have not been ascribed to a specific bioactive compound, it is believed that the synergy among the plant bioactive compounds contributes to its complex mechanism of action. *L*-canavanine, D-pinitol, and GABA are reported as the most bioactive elements within the plant.

The following sections will discuss a more detailed exploration of the biological activity of different organic extracts and bioactive compounds.

### 7.1. Cancer

The ethanolic extract of *L. frutescens* has demonstrated a significant cytotoxic effect on normal T-lymphocytes, particularly at a concentration of 2.5 mg/mL. After 24 h, the extract induced necrosis in 95% of cells, depleted ATP levels by 76%, and inhibited caspase 3/7 activity by 11%. In contrast, the deionised water extract at the same concentration caused milder effects, with necrosis at 26%, ATP levels at 91%, and caspase 3/7 inhibition at 15%. Both extracts exhibited time-dependent effects over 48 h, with the ethanolic extract showing more potent inhibition of cell growth through necrosis, ATP depletion, and reduced caspase activity. DNA fragmentation observed after 48 h confirmed the potential toxicity of the extracts, although the water extract appeared relatively safer [57].

**Figure 5:**
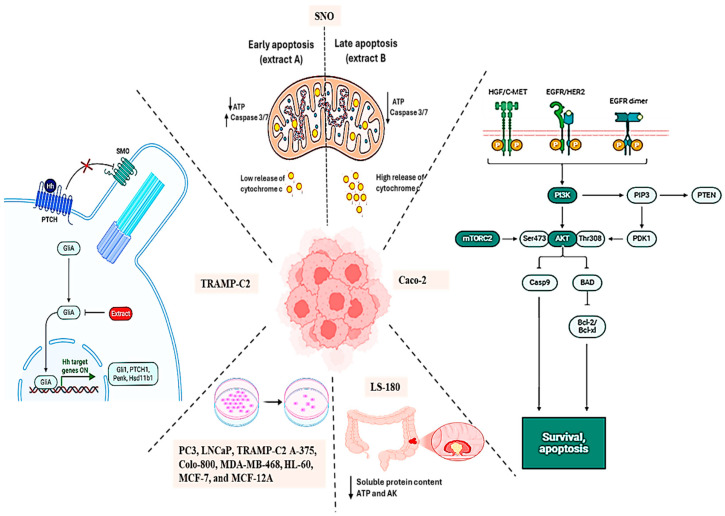
Molecular pathways modulated by *L. frutescens* extracts: Hedgehog, mitochondrial, and PI3K/AKT signalling in cancer models. Created using BioRender.com [67].

Ethanolic extracts of *L. frutescens* have also shown anticancer activity. Tai et al. [9] reported that the ethanolic extract inhibited the proliferation of cancer cell lines, including Jurkat, MDA-MB-468 (malignant breast cancer), HL-60 (human leukaemia), and MCF-7 (breast cancer) with IC_50_ values of 0.91 mg/mL (1/150 dilutions), 0.68 mg/mL (1/200 dilutions), 0.68 mg/mL (1/200 dilutions), and 0.55 mg/mL (1/250 dilutions), respectively. The active compound *L*-canavanine, a non-proteinogenic amino acid, was implicated in the antiproliferative effects by inhibiting enzyme function and inducing protein misfolding [65]. Interestingly, *L*-arginine at 1 mM mitigated the antiproliferative effects of 2 mM *L*-canavanine in MCF-7 cells, suggesting a potential pathway to modulate toxicity. Further studies by Stander et al. [40] showed that a 70% ethanolic extract of *L. frutescens* (1.5 mg/mL) inhibited MCF-7 cell proliferation and induced apoptosis within 72 h. An aqueous extract at 10 mg/mL reduced cell growth by 26% in MCF-7 cells and 49% in MCF-12A cells. In MCF-7 cells, pronounced apoptotic changes, such as chromatin condensation and apoptotic bodies, were observed. Flow cytometry revealed a heightened sub-G1 apoptotic fraction and S-phase arrest. Transmission electron microscopy suggested that these effects were driven by autophagic and apoptotic processes, likely induced by *L*-canavanine’s protein misfolding response [54]. In contrast, Steenkamp and Gouws [66] reported that an aqueous extract (50 µg/mL) exhibited minimal cytotoxicity against MCF-7, DU-145 (prostate cancer), MDA-MB-231, and MCF-12A cells, suggesting that concentrations ≤ 50 µg/mL, the plant does not exhibit antiproliferative properties (Figure 5).

Methanolic extracts of *L. frutescens* have demonstrated cytotoxic effects against prostate cancer cell lines PC3, LNCaP, and TRAMP-C2, with IC_50_ values of 167, 200, and 100 µg/mL, respectively. These effects were independent of androgen receptor signalling and involved suppression of Gli/Hh signalling, as evidenced by reduced Gli1 and Ptch1 gene expression, which plays a role in prostate cancer tumorigenesis [10]. Similarly, ethanolic extracts have been shown to downregulate PI3K/Akt signalling, reduce FKHR phosphorylation, and activate mitochondrial apoptotic pathways in Caco-2 colon cancer cells, promoting apoptosis [39]. The aqueous extract at 2.63 mg/mL further induced cytotoxicity in LS180 colorectal cancer cells, depleting soluble protein content, intracellular ATP, and extracellular adenylate kinase within 24 h [41].

In melanoma and cervical cancer models, ethanolic extracts reduced the viability of melanoma cells (A-375 and Colo-800) by 62% and 43%, respectively, after 72 h at 0.625 mg/mL. It showed even greater efficacy against human dermal fibroblast cells (HDFα), where viability decreased by 81% at 0.3 mg/mL after 72 h [36]. Meanwhile, it was reported that the aqueous extract at 3.5 mg/mL induced apoptosis and cytotoxicity in Chinese hamster ovary (CHO) and cervical neoplastic cells [56]. Studies on oesophageal cancer (SNO cells) highlighted geographical variations in extract efficacy. Extracts from Colesberg induced apoptosis through caspase 3/7 activation, while extracts from Platvei triggered cytochrome c release, highlighting the influence of geographical variations on the phytochemical composition and biological activity of the plant [38].

**Figure 6:**
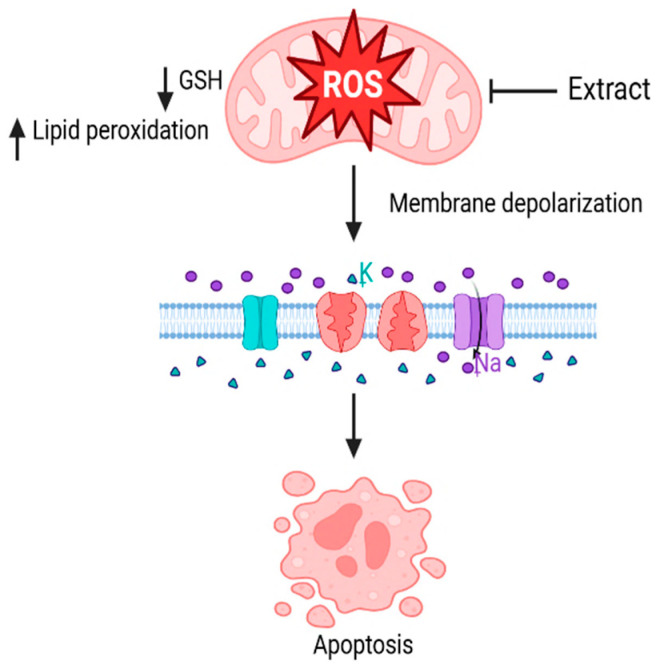
Cytotoxic effects of *L. frutescens* on renal epithelial cells: oxidative stress and mitochondrial dysfunction. Created using BioRender.com [67].

Phulukdaree et al. [56] reported that *L. frutescens* aqueous extract (6 mg/mL) significantly reduced intracellular glutathione levels, increased lipid peroxidation, and induced mitochondrial membrane depolarisation (in 80% of the treated cells) in MDBK and LLC-PK1 cells. At higher concentrations (12 and 24 mg/mL), the extract increased oxidative stress, disrupted mitochondrial integrity, and promoted apoptosis. These findings suggest that *L. frutescens* aqueous extract has dose-dependent cytotoxic effects on MDBK and LLC-PK1 cells, mediated primarily through the induction of oxidative stress and mitochondrial damage (Figure 6).

### 7.2. COVID-19

A molecular docking investigation revealed that *L*-canavanine is a promising inhibitor of the SARS-CoV-2 3CL^Pro^, showing favourable binding modes and strong interactions in the active site of 3CL^Pro^ [68]. Moreover, Akindele et al. [69] also reported that apart from its antiviral, anti-inflammatory, and immunomodulatory properties, the plant also possesses COVID-19 symptom-relieving activity.

### 7.3. Antioxidant

The ethanolic extract of *L. frutescens* demonstrated significant hydroxyl radical scavenging in the TEAC assay but failed to modulate LPS-induced NO production in RAW 264.7 cells across various concentrations ranging from 0.068 to 0.68 mg/mL. In contrast, *L*-canavanine (0.5 mM) and D-pinitol (10 mM) significantly inhibited LPS-induced NO secretion. Given *L*-canavanine’s role as a selective inhibitor of iNOS, the absence of inhibitory activity by *L. frutescens* may be concentration-dependent [9]. Similarly, Fernandes et al. [42] reported that a hot aqueous extract of *L. frutescens* at a concentration of 10 µg/mL reduced the luminol- and lucigenin-enhanced chemiluminescence response in FMLP-stimulated neutrophils. In the hydrogen peroxide/horseradish peroxidase-mediated chemiluminescence, it scavenged neutrophil-derived oxidants at 2.5 µg/mL. Furthermore, at a concentration of 0.62 µg/mL, it inhibited horseradish peroxidase/hydrogen peroxide-induced chemiluminescence [42]. Moreover, the antioxidant efficiency of *L. frutescens* varied significantly depending on the extraction solvent [44]. This variation is primarily attributed to the solvent’s impact on the composition of phenolics and flavonoids in the extract. Solvents with higher polarity yielded extracts with greater total phenolic and flavonoid content, resulting in greater reducing power and radical scavenging activity [44,70]. The freeze-dried hot water extract of *L. frutescens* (500 µg/mL) demonstrated protective effects against tert-butyl hydroperoxide (t-BHP)-induced oxidative stress in CHO, human hepatoma (HepaRG), and human pulmonary alveolar carcinoma (A549) cells by effectively scavenging ROS and preserving intracellular glutathione (GSH/GSSG) levels. At a 1 mg/mL concentration, the extract exhibited potent scavenging activity, effectively neutralising hydroxyl radicals, followed by superoxide radicals and hydrogen peroxide [44].

### 7.4. Immune Modulation and Inflammation

**Figure 7:**
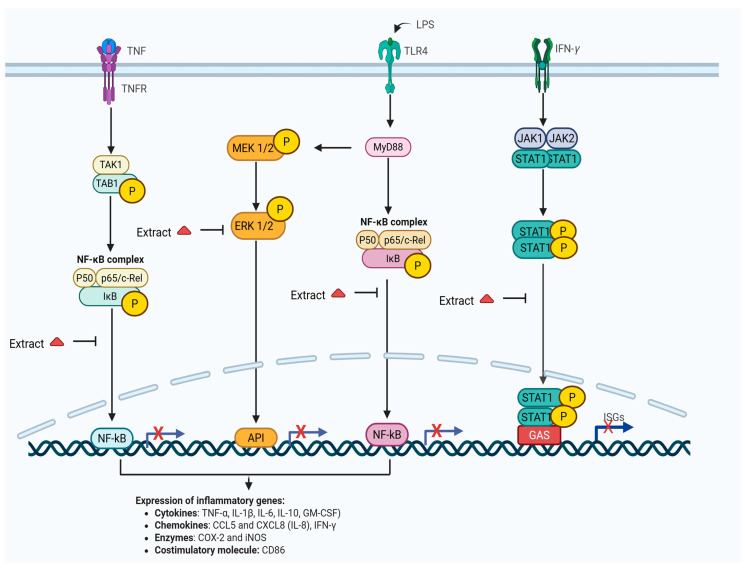
Inhibitory effects of *L. frutescens* extracts on inflammatory signalling pathways. Created using BioRender.com [67].

Na et al. [71] reported that the methanolic extract (10, 5, and 1 µg/mL) of *L. frutescens* inhibited 12-*O*-tetradecanoylphorbol-13-acetate (TPA)-induced cyclooxygenase-2 (COX-2) expression in human breast epithelial (MCF10A) cells by suppressing the DNA-binding activity of nuclear factor kappa-light chain enhancer of activated B cells (NF-κB) induced by TPA (10 nM). This inhibition of TPA-induced COX-2 expression, achieved through suppressing NF-κB DNA binding, may be responsible for the plant’s chemopreventive activity [71]. The aqueous extract also partially reduced tumour necrosis factor-alpha (TNF-α) induced chemokine CCL5 expression in NRK-52E cells [46]. The transcriptome analysis has provided valuable insights into the role of *L. frutescens* in modulating the immune system. The crude polysaccharide-enriched fraction of *L. frutescens* aqueous extract influenced gene expression in activated murine macrophage cell lines (RAW 264.7), resulting in the differential expression of 547 genes [20,51]. This fraction also exhibited immuno-stimulatory activity by activating macrophages via TLR4 receptors and the NF-κB signalling pathway [51]. Additionally, the ethanolic extract-enriched polysaccharides fraction reduced the production of nitric oxide (NO) and reactive oxygen species (ROS), as well as inhibited the phosphorylated extracellular signal-regulated kinase ½ (p-ERK1/2), signal transducer, and activator of transcription 1-α (STAT1-α) and NF-κB induced by lipopolysaccharides (LPS) and interferon-gamma (IFNγ) [45]. Furthermore, the ethanolic and aqueous extracts were found to significantly inhibit GM-CSF, G-CSF, IL-1α, IL-6, TNF-α, iNOS, NO, ROS, COX-2, and CD86 (Figure 7). The ethanolic extract also modified the M1 and M2 macrophage phenotypes’ expressions by enhancing the M2 phenotype and downregulating the M1 phenotype [50]. These findings corroborate the results of Lei et al. [45] regarding the plant anti-inflammatory macrophage markers.

*L. frutescens* was also shown to negatively regulate the NF-κB signalling pathway by suppressing NF-κB nuclear translocation following LPS induction. This further substantiates the plant’s ability to inhibit NF-κB activation through the attenuation of NF-κB p65 subunit phosphorylation on the Ser 536 residue, which is essential for both NF-κB nuclear transcriptional and translocation activity [50]. Likewise, Kirsten [72] stated that the plant extract regulated the expression of IL-6, IL-10 and IFN-γ in phytohaemagglutinin (PHA) and LPS. However, it was reported that these immune modulation effects are donor-dependent [72]. Additionally, little effect of the ethanol extract on the stimulation of TNF-α and IL-8 by phorbol myristoyl acetate was observed [43,73]. Jiang et al. [43] further demonstrated that the ethanol extract could mitigate N-methyl-D-aspartic acid (NMDA) induced neuronal oxidative responses and reduce ROS and NO production induced by LPS and IFN-γ in microglial cells (BV-2 and HAPI).

Moreover, the ability of the ethanolic extract to inhibit IFN-γ-induced p-ERK1/2 pathway explains the extract’s potential in preventing or treating inflammatory infections, including HIV-associated neurocognitive disorders. *L. frutescens* shoot aqueous extract inhibited fresh egg albumin-induced acute inflammation, triggering hypoglycemia in rats [3,69]. Its efficacy as an antidiabetic and anti-inflammatory herbal remedy can be attributed to its inhibitory effects on cytokines and apoptosis [64,74,75]. Furthermore, it also found that the aqueous extract inhibited the gene expression of CYP3A4 and CYP2D6 enzymes instead of inducing them [41].

### 7.5. Nephrotoxicity

The sugar D-pinitol improved histopathological alterations in cisplatin-induced nephrotoxicity in mice because of its antiapoptotic, antioxidant, and anti-inflammatory properties [64]. Likewise, the reduction in histopathological and biochemical alterations, as well as decreasing levels of cytokines (TNF-α, IL-6, and IL-1β) and oxidative stress in cisplatin-induced nephrotoxicity, ameliorate the nephrotoxic reaction of cisplatin in D-pinitol-treated mice [64].

### 7.6. Antimicrobial

The IC_50_ of *L. frutescens* hexane extract against *Enterococcus faecali*, *Escherichia coli*, and *Staphylococcus aureus* were found to be 2.50, 1.25, and 0.31 mg/mL, respectively [60]. Conversely, the dichloromethane–methanol (1:1) extract displayed a good inhibition against the shikimate kinase enzyme, an important drug target for *Mycobacterium tuberculosis,* with an IC_50_ of 0.1 μg/mL, whereas the aqueous and ethanolic extracts had IC_50_ of 5.1 and 1.7 μg/mL, respectively [11]. The efficiency of the dichloromethane–methanol (1:1) extract as shikimate kinase inhibitor was attributed to the essential omega-3 fatty acid α-linolenic acid, which is known for its antimicrobial activity against *Staphylococcus aureus*, *Bacillus subtilis*, *Helicobacter pylori*, *Rhizoctonia solani*, *Crinipellis perniciosa*, and hepatitis C virus [11,75,76,77,78]. α-linolenic acid was found to possess antitubercular activity by inhibiting the shikimate kinase enzyme with an IC_50_ of 3.7 μg/mL [11].

The ethyl acetate and 50% methanolic extracts were diluted to 5%, 10%, and 20% (*w*/*w*) with DMSO and tested for their mutagenic and antimutagenic properties against *Salmonella typhimurium* strains TA97a, TA98, TA100, and TA102. After investigations, it was observed that the ethyl acetate extract significantly exhibited antimutagenic effects against TA97a, TA98, TA100, and TA102 [58]. On the other hand, the methanolic extract showed pro-mutagenic and antimutagenic potential in the presence of the S9 in TA98 with 2-acetamidofluorene and TA100 with aflatoxin B1. *L*-arginine, GABA, and D-pinitol exhibited antimutagenic activity against all four strains, whilst *L*-canavanine displayed a co-mutagenic effect in the absence of S9 in TA97 with 9-aminoacridine. Thus, the pro-mutagenic activity of the methanol extract cannot be ascribed to *L*-canavanine [58]. The ethyl acetate extract, having a higher antimutagenic potential and total phenolic content than the methanolic extract, explains the correlation between the antioxidant and antimutagenic activities of the plant.

### 7.7. HIV/AIDS

**Figure 8:**
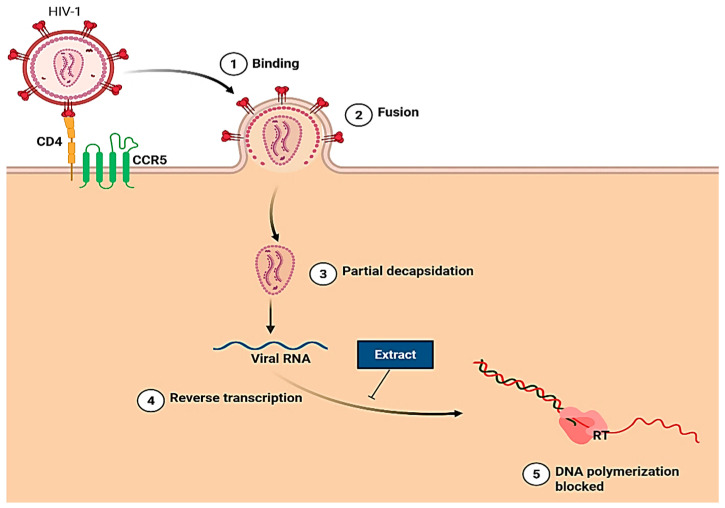
Mechanism of action of *L. frutescens* on HIV-1 reverse transcriptase. Created using BioRender.com [67].

The aqueous extract (200 µg/mL) of the leaves was reported to inhibit the HIV-1 reverse transcriptase (RT) enzyme (Figure 8) by ≥50% [47]. However, when tested with 0.2% (*w*/*v*) bovine serum albumin (BSA) to neutralise tannin effects, the inhibitory activity was reduced, indicating that tannins contributed significantly to the inhibition. Despite this, the extract retained approximately 30% of its activity. On the other hand, the dichloromethane extract exhibited limited activity against the HIV-II protease enzyme but significantly inhibited α- and β-glucosidase enzymes [47].

An ethanolic extract concentration equivalent to its calculated IC_50_ (7.5 mg/mL) was administered to normal human lymphocytes for 3, 6, and 12 h. At the 12 h mark, the extract induced apoptosis in total lymphocytes, with a stronger effect on CD4^+^ subpopulations. This was supported by increased caspase-3/7 activity, phosphatidylserine (PS) translocation, and reduced ATP levels [79]. Additionally, after 12 h, the extract doubled the number of lymphocytes expressing the CD69 activation marker, leading to activation-induced cell death. These findings contradicted earlier clinical suggestions that the extract might be useful in treating HIV/AIDS [79].

D-pinitol and GABA have been proposed to alleviate wasting conditions in cancer and HIV/AIDS patients by inhibiting inflammatory cytokines TNF-α and IL-1β, thus enhancing glucose availability for cell metabolism [79,80,81]. Conversely, *L*-canavanine demonstrated antiviral properties against HIV and influenza by disrupting viral protein synthesis and function [79,82]. Chronic oral administration of *L. frutescens* extract (12 mg/kg for 5 days) induced intestinal and hepatic CYP3A2 expression in rats, altering the pharmacokinetics of the antiretroviral drug nevirapine and increasing CYP3A4 activity in LS180 cells [62]. This suggests a potential drug–herb interaction when the nevirapine is co-administered with *L. frutescens*. D-pinitol and the aqueous extract reduced atazanavir accumulation in Caco-2 cells at 10 mg/mL, potentially lowering its bioavailability, while a triterpenoid glycoside-enriched fraction enhanced atazanavir accumulation and absorption [52]. Additionally, the methanolic and aqueous extracts of *L. frutescens* inhibited atazanavir metabolism in human liver microsomes, indicating a potential impact on the drug’s clinical metabolism and absorption [52].

**Figure 9:**
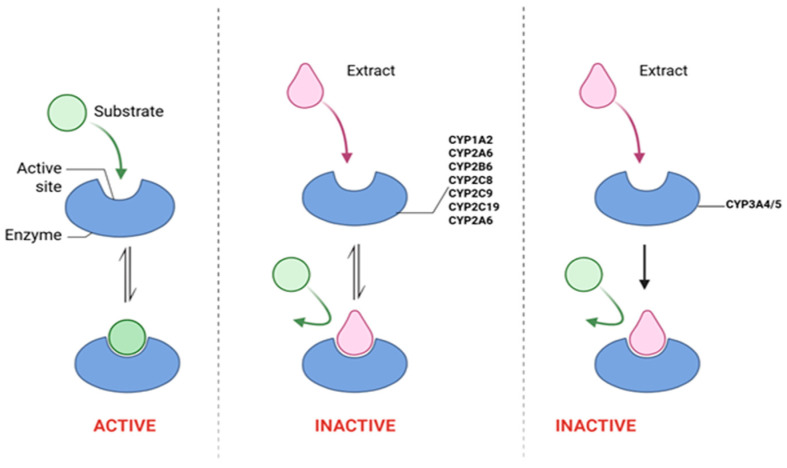
Inhibition of P450 enzyme activity. Created using BioRender.com [67].

Fasinu et al. [53] also demonstrated the anti-HIV activity of *L. frutescens* against various cytochrome P450 isozymes. These include CYP1A2-mediated phenacetin demethylation, CYP2A6-mediated coumarin 7-hydroxylation, CYP2B6-mediated bupropion hydroxylation, CYP2C8-mediated paclitaxel 6α-hydroxylation, CYP2C9-mediated diclofenac 4′-hydroxylation, CYP2C19-mediated *S*-mephenytoin 4′-hydroxylation, CYP3A4/5-mediated midazolam 1′-hydroxylation, and CYP3A4/5-mediated testosterone 6β-hydroxylation in pooled human liver microsomes (Figure 9) with IC_50_ values of 41, 160, 20, 22.4, 23, 35.9, 17.5, and 28.3 μg/mL, respectively [53]. The studied extract induced time-dependent (irreversible) inhibition of CYP3A4/5 with an inhibition constant (Ki) of 296 μg/mL and a maximal rate of enzyme inactivation (Kinact) of 0.063 min^−1^ [53]. The authors also indicated that the plant inhibited the human ATP-binding cassette transporters P-gp as well as the organic anion transport polypeptide OATP1B1 and OATP1B3 with IC_50_ values of 324.8, 10.4, and 6.6 μg/mL, respectively. This inhibition also led to a 40% reduction in the clearance of midazolam metabolites in hepatocytes. However, no activity was observed when treating the efflux transporter BRCP (breast cancer resistance protein) as well as the enzymes CYP2D6 and CYP2E1 with *L. frutescens* [53].

**Figure 10:**
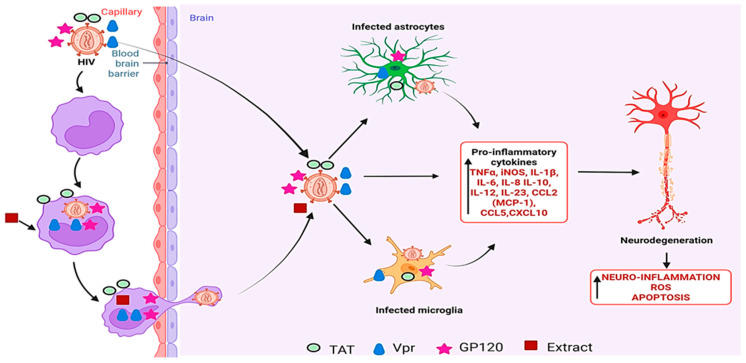
Impact of *L. frutescens* extracts on HIV-induced neuroinflammation. Created using BioRender.com [67].

Despite some therapeutic potential, *L. frutescens* also raised safety concerns. Africa and Smith [83] found that the plant significantly reduced IL-1β secretion but increased monocyte chemoattractant protein-1 (MCP-1) levels, leading to greater infiltration of CD14^+^ monocytes across the blood–brain barrier (Figure 10). This exacerbated HIV-associated neuroinflammation, prompting warnings against its use by HIV patients at any stage of infection [83,84].

### 7.8. Neuroprotection

Pre-treatment of 1-methyl-4-phenylpyridinium (MPP^+^) induced toxicity in SH-SY5Y neuroblastoma cells, with the plant aqueous extract resulting in the protection of the cells from the MPP^+^ induced toxicity and loss of MPP via the regulation of ROS, thus hinting at the extract’s neuroprotective effect and its potential as an anti-Parkinson agent [49]. In a study by Ndjoubi et al. [13], several natural compounds, including 8-methoxyvestitol; mucronulatol; proline; D-pinitol; sutherlandin C; sutherlandiosides B, D, K; and 7*S*,24*S*,25-trihydroxy-9,10*R*-seco-9,19-cyclolanost-2(3),9(11)-diene-25-*O*-*β*-D-glucopyranoside, demonstrated significant neuroprotective effects through their antiapoptotic activity. The compounds were evaluated for their antiapoptotic potency, with sutherlandioside B, mucronulatol, proline, and D-pinitol significantly restoring ATP levels from 51% (MPP^+^-treated) to 73, 75, 74, and 75%, respectively, while inducing caspase 3/7 activity from 5 fold to 1.5–2.8 fold relative to controls, with mucronulatol exhibiting the most potent antiapoptotic effect (1.5-fold) [13].

### 7.9. Diabetes

Studies have shown the effectiveness of *L. frutescens* extracts in managing diabetes through various mechanisms. Oral administration of the shoot aqueous extract strongly inhibited streptozotocin-induced hyperglycemia in mice at concentrations ranging from 50 to 800 mg/kg [3]. Additionally, the aqueous leaf extract showed promise as a type 2 antidiabetic drug by significantly increasing glucose uptake into muscle and adipose tissue while significantly decreasing intestinal glucose uptake (after 1 h). This indicates the extract’s potential to normalise insulin levels and glucose uptake in peripheral tissues and suppress intestinal glucose uptake without causing weight gain [20,61]. Studies on rats fed on a high-fat diet showed that the plant extracts prevent the development of insulin resistance by reducing plasma-free fatty acid levels [85]. It was also observed that rats on a high-fat diet exhibited a twelve-fold reduction in plasma-free fatty acid levels compared to those on a normal diet [85]. Moreover, Bates et al. [86] stated that D-pinitol acted similarly to insulin by lowering blood sugar levels and augmenting glucose uptake for cellular metabolism. This resulted in its capacity to regulate cellular energy by boosting energy levels and reducing fatigue. In 2013, it was discovered that the aqueous extract could prevent insulin resistance in hepatocytes [48].

### 7.10. Stress

The warm water extract of *L. frutescens* leaves was revealed to efficiently reduce the corticosterone response to chronic stress in Wistar rats [59]. This finding confirmed the traditional use of the plant in treating ailments associated with high levels of glucocorticoids. Investigations on the aqueous and methanolic extracts revealed these extracts inhibit progesterone (PROG) binding to CYP17A1 and CYP21A2 without affecting 3β-HSD2 [59]. The methanolic extract containing sutherlandioside B (SUB) as its major component significantly inhibited pregnenolone (PREG)and PROG conversion by CYP17A1. Interestingly, at lower concentrations, the extract could considerably affect the catalytic activity of CYP17A1 only by PROG conversion [12]. The absence of an inhibitory effect on PREG metabolism suggests that the plant’s bioactive compounds may bind to a site in the active pocket other than the one occupied by PREG [59]. Changes observed in the inhibition of PROG and PREG metabolism and substrate binding imply that the extract’s bioactive components probably act synergistically and interfere with the electron transport chain to inhibit CYP17A1 and CYP21A2 enzymes [59]. Furthermore, SUB was reported to inhibit CYP17A1 towards PREG and PROG as well as 3β-HSD2, signifying that SUB could disrupt steroidogenesis at the branch point [12]. In human H295R adrenal cells, the extract inhibited CYP11B1 by considerably reducing cortisol (CORT) and 11-hydroxy androstenedione (11-OHA4) levels, explaining the plant’s antistress, anti-anxiety, and anti-hypertensive properties [12]. Moreover, the methanol extract and SUB acted as selective glucocorticoid receptor agonists (SEGRAs) by not showing any transactivation ability on glucocorticoid response element-driven gene expression. SUB and the studied plant extract also suppressed NF-κB -driven gene expression while being unable to activate mineralocorticoid receptor (MR) mediated gene transcription, although both antagonised the effects of aldosterone via MR [12].

The non-protein free amino acid GABA exhibits anti-neurotransmitter properties, which partly explain the use of *L. frutescens* for stress and anxiety disorders [36]. The anti-anxiety activity of GABA has been linked to its ability to reduce glucocorticoid production [80].

### 7.11. Toxicology

The traditional dosage involves daily infusions or decoctions of 2.5–5 g of dried material. The highest recorded dose, a decoction of 5 g of leaves, stems, and pods taken twice daily over six years, resulted in no adverse effects [4]. Furthermore, studies on the intraperitoneal administration of graded aqueous extracts of *L. frutescens* in fasted Balb C albino mice (20–25 g) established the lethal dose (LD_50_) at 1280 ± 71 mg/kg, suggesting that the crude extracts are likely to be relatively safe in mammals [3]. A study on determining the toxicity of the aqueous and ethanolic leaf extracts on zebrafish embryos, focusing on their hatching rates and larval mortality at concentrations ranging from 5 to 300 µg/mL, exhibited lethal concentration (LC_50_) values of 297.57 µg/mL (aqueous) and 40.54 µg/mL (ethanol), reaffirming the claim that the water extract is less toxic than the ethanol extract [84]. However, further study on how the plant may interact with other drugs and diseases is essential to avoid fatal or detrimental side effects.

For commercial preparations, a recommended dose of 300 mg of dried leaves twice daily (600 mg/day) is advised, with the caution that it should be avoided during pregnancy and lactation. This conservative dosage was used for a safety study in vervet monkeys, where doses of 0, 9, 27, and 81 mg/kg body weight correspond to 0, 1, 3, and 9 times the recommended human dose. These doses were administered as part of a standard diet for three months, and the study showed no clinical side effects across 15 haematological, 21 clinical biochemical, 6 physiological, and many behavioural variables, providing strong and reassuring evidence of the safety of *L. frutescens* at recommended human doses [4].

### 7.12. Clinical Trials

**Figure 11:**
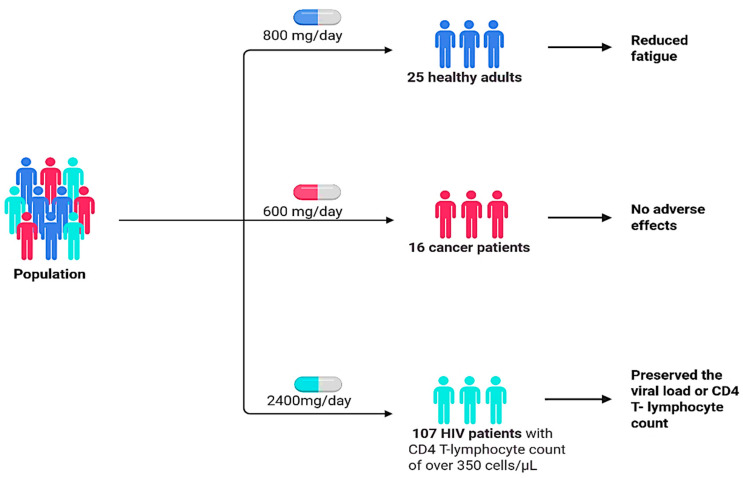
*L. frutescens* clinical trials outcomes. Created using BioRender.com [67].

Grandi et al. [87] conducted a study with 16 cancer patients (11 men and 5 women) to evaluate the effect of 600 mg/day of aqueous *L. frutescens* extract (Figure 11). They found that the extract significantly decreased fatigue in cancer patients, with no other major adverse effects reported (Table 4, Figure 10). In Johnson et al. [5], a randomised, double-blind, placebo-controlled trial involving 25 healthy adults examined the effects of 800 mg/day of *L. frutescens* leaf powder capsules. The study concluded that the powder was well tolerated over three months, with no significant adverse events observed, and there was a noted improvement in appetite in the treatment group [5]. In 2002, the South African Ministry of Health recommended the aqueous extract as a drug support in the treatment of HIV/AIDS [8] as it decreased viral loads and improved CD4 counts [88]. However, preclinical studies performed in 2011 have indicated that using *L. frutescens* extract alongside antiretroviral drugs or CYP3A4 substrates may cause harmful drug–herb interactions, treatment failure, and the development of viral resistance [8,62]. Wilson et al. [89] performed a study on 107 participants, dividing them into two groups: one received 2400 mg/day of *L. frutescens* leaf powder (1200 mg twice daily), and the other received a placebo. The results showed that *L. frutescens* did not alter the viral load or CD4 T-lymphocyte count, but the treatment group had a higher burden, primarily due to two tuberculosis cases in patients on isoniazid preventive therapy (IPT). While no other safety concerns related to *L. frutescens* consumption were detected, the study indicates the need for further investigation into the potential interaction between *L. frutescens* and IPT [89].

## 8. Discussion

*L. frutescens* has been extensively studied for its anticancer, anti-inflammatory, immune booster and anti-HIV properties, with traditional usage suggesting minimal side effects. However, research studies highlight significant complexities and limitations in its application, particularly in anticancer and anti-HIV therapies, while revealing promise in other areas such as immune booster, metabolic, oxidative, and microbial conditions.

The cytotoxic effects of *L. frutescens* extracts have been demonstrated in various cancer cell lines. These effects are primarily mediated through mechanisms such as PI3K/Akt inhibition, oxidative stress, mitochondrial dysfunction, apoptosis, caspase activation, and suppression of the Gli/Hh signalling pathway. Despite these promising results, the therapeutic concentrations required (0.3–10 mg/mL) are significantly higher than those of standard chemo-therapeutic drugs like doxorubicin (IC_50_ = 0.68 ± 0.04 μg/mL for MCF-7) and paclitaxel (IC_50_ = 2.5 ng/mL for MCF-7 and 2.6 ng/mL for HeLa), raising concerns about the practical application of *L. frutescens* in cancer therapy [90,91]. Additionally, the lack of selectivity is a major limitation, as both cancer and normal cells (T-lymphocytes) exhibit toxicity at similar concentrations. This raises questions about the plant’s therapeutic index and clinical safety for cancer patients.

In HIV therapy, *L. frutescens* has shown inhibitory effects on HIV-1 RT, primarily due to its tannin content. However, tannins lack specificity, and their pharmacological efficacy is significantly lower than the established antiretroviral drugs [92]. For instance, tenofovir, a nucleotide analogue RT inhibitor, exhibits highly specific activity by competing with the natural substrate, deoxyadenosine 5′-triphosphate, for incorporation into viral DNA at the active site of reverse transcriptase, ultimately causing premature termination of the DNA chain during replication with IC_50_ values of 0.5–2.2 µM with a highly specific mechanism of action [93,94,95]. On the other hand, *L. frutescens* extracts require much higher concentrations (IC_50_ = 200 µM), limiting their practical utility.

Furthermore, the immunotoxicity of *L. frutescens* further complicates its potential use in HIV therapy. At an IC_50_ concentration of 7.5 mg/mL, the ethanolic extract induces apoptosis in lymphocytes and CD4^+^ cells by increasing caspase-3/7 activity, promoting phosphatidylserine translocation, and depleting ATP levels. This activation-induced cell death significantly undermines the preservation of the CD4^+^ T-cell population, a critical goal in HIV treatment [96]. The resulting loss of immune integrity heightens susceptibility to opportunistic infections, such as tuberculosis and fungal infections, presenting a substantial drawback for therapeutic application.

Its potential for drug–herb interactions poses significant challenges, as it may compromise the efficacy and bioavailability of antiretroviral drugs, creating risks for patients who require precise drug concentrations. Additionally, *L. frutescens* has been associated with exacerbating HIV-associated neuroinflammation, evidenced by increased MCP-1 levels and CD14^+^ monocyte infiltration across the blood–brain barrier. These effects could worsen HIV-associated neurocognitive impairment, a condition affecting 42.6% of patients [96,97]. Despite the higher concentrations required for anticancer and anti-HIV activity, *L. frutescens* has demonstrated more promising potential in other therapeutic areas. Studies highlight significant antidiabetic, antioxidant, neuroprotective, anti-inflammatory, and antimicrobial properties. Both aqueous and ethanolic extracts, along with bioactive compounds such as sutherlandioside B, mucronulatol, D-pinitol, and α-linolenic acid, exhibit potent biological activity at concentrations below 50 µg/mL (Table 2 and Table 3). For instance, D-pinitol is recognised for its insulin-sensitising effects, while α-linolenic acid, sutherlandioside D, and mucronulatol contribute to the plant’s anti-tubercular, antistress, and neuroprotective activities, respectively. Such properties underscore the plant’s potential in addressing metabolic and oxidative stress-related disorders.

## 9. Economic Importance of *L. frutescens*

**Figure 12:**
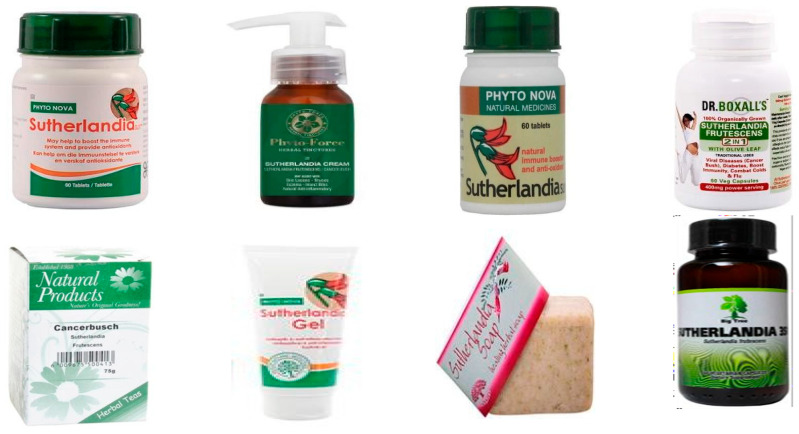
The different forms of *L. frutescens* in the market (Pictures sourced from the South African local market).

In South Africa, the commercial value of *L. frutescens* lies in its renowned ethnopharmacological applications, particularly in treating internal cancers, HIV/AIDS, and diabetes. Additionally, it is prized for its immune-boosting and antioxidant properties and its use as a skincare product. Over the past decade, the plant has been sold in various forms, including capsules, tablets, teas, syrup, soap, cream, and raw materials products, as shown in Figure 12.

Moreover, the global trade of *L. frutescens* extends to regions such as North and South America, Western and Eastern Europe, Asia-Pacific, the Middle East, and Africa, with Africa and the Middle East holding a significant share of the global trade. Different South African companies are leading retailers and distributors of the various processed and semi-processed forms of *L. frutescens* [98].

Its popularity in traditional medicine has sparked significant scientific interest in understanding the pharmacokinetics and pharmacodynamics of *L. frutescens* crude extracts and identifying the specific metabolites responsible for its ethnobotanical properties and its ability to treat conditions related to oxidative stress. These investigations have prompted companies and researchers to patent their formulations and extraction methods for sale [99,100].

## 10. Conclusions

The phytochemical investigation on *L. frutescens* has highlighted its potential as a rich source of amino acids, flavonoid glycosides, and cycloartane triterpenes glycosides. The discovery of novel cycloartane glycosides and 9,10-seco-cycloartane-type diglycosides with an unprecedented 5/7/6/5 ring skeleton highlights their significance in natural product chemistry. Moreover, the oxygenation pattern of rings A and C of many of the isolated compounds is unique and limited to this plant.

Despite its promising bioactivity, the therapeutic efficiency of *L. frutescens* faces challenges, including its high effective concentrations, lack of selectivity, potential drug–herb interactions, and immunotoxicity. Most observed activities occur at concentrations exceeding the 50 µg/mL threshold, raising concerns about its clinical relevance for cancer and HIV treatment. Nonetheless, its potential in managing diabetes, neurodegenerative disorders, microbial infections, and oxidative-related conditions presents an opportunity for further exploration.

## Figures and Tables

**Table 1:** The IUPAC names of the sutherlandiosides E-H isolated by Fu [33] and Tchegnitegni et al. [34].

Compounds	Fu [33]	Tchegnitegni et al. [34]
Sutherlandioside E	24,25-*O*-*β*-D-diglucopyranosyl-3*S*, 24*S*,25-trihydroxy, 2(1-10)-abeo-9, 10*R*-seco-cycloartanoic acid (**20**)	7*S*,24*S*,25-trihydroxy-9, 10*R*-*seco*-9,19-cyclolanost-2(3), 9(11)-diene-25-*O*-*β*-D-glucopyranoside (**22**)
Sutherlandioside F	24,25-*O*-*β*-D-diglucopyranosyl 3*R*, 24*S*,25-trihydroxy, 2(1-10)-abeo-9, 10*R*-seco-cycloartanoic acid (**21**)	1*S*,3*R*,7*S*,24*S*,25-pentahydroxycycloartan-11-one-25-*O*-*β*-*D*-glucopyranoside (**23**)
Sutherlandioside G	(3*R*,7*S*,24*S*,25)-[3-*O*-*β*-D-glucopyranosyl]-tetrahydroxycycloartan-1-one-25-*O*-*β*-*D*-glucopyranoside (**18**)	1*S*,3*R*,7*S*,24*S*,25-pentahydroxycycloartan-11-one-24-*O*-*β*-D-glucopyranosyl-25-*O*-*β-D*-glucopyranoside (**24**)
Sutherlandiosde H	3*R*,24*S*,25-trihydroxycycloartane-1,11- dione-24, 25-*O*-*β*-D-diglucopyranoside (**19**)	3*R*,24*S*,25-trihydroxycycloartan-1-one-25-*O*-*β*-D-glucopyranoside (**25**)

**Table 2:** Ethnopharmacological and pharmacological properties of *L. frutescens* extracts, fractions, and compounds in in vitro studies (whole plant extracts considered when specific plant parts are not mentioned).

Pharmacological Activity	Extract/Compound	Experimental Models	Tested Concentrations	Experimental Outcomes	References
Anticancer	70% ethanol (tablet)	MDA-MB-468, HL-60, MCF-7, and Jurkat cells	IC_50_: 0.91–0.55 mg/mL	Inhibited growth of MCF7 (0.55 mg/mL), MDA-MB-468 (0.68 mg/mL), Jurkat (0.91 mg/mL), and HL60 (0.68 mg/mL)	[9]
		A-375, Colo-800 cells cells	0.625 mg/mL	Inhibited proliferation	[36]
		HDFα cells	0.3 mg/mL	Reduced HDFα viability to 19% after 72 h	
	70% ethanol	PC3, LNCaP, and TRAMP-C2 cells	100–200 µg/mL (IC_50_)	Suppressed the growth of PC3 (167), LNCaP (200), and TRAMP-C2 (100) while inhibiting Gli/Hh signalling activity by downregulating Gli1 and PTCH1 gene expression in TRAMP-C2 and PC3 cells	[10]
		SNO cells	2.5 and 5 mg/mL	Reduced ATP levels; apoptotic effect; decreased caspase 3/7 levels	[38]
		CaCo-2 cells	2.73 mg/mL	Inhibited the PI3-K/Akt pathway by decreasing the phosphorylation of p85, p110, Akt (Ser473 and Thr308), and PTEN Promoted apoptosis by increasing PARP cleavage and cleaved caspase-9 levels while significantly reducing total Bax and c-IAP levels	[39]
	70% ethanolic (leaves and twigs)	MCF-7 cells	1.5 mg/mL	Inhibited the proliferation of MCF-7 cells after 72 h of exposure	[40]
	Sutherlandioside B and D	Shh Light II cells	10 µg/mL	Inhibited Gli-reporter activity by 22% and 89%, respectively	[10]
	Aqueous	LS180 cells	2.63 mg/mL (IC_50_)	Reduced cell growth, viability, ATP, and AK levels relative to protein content	[41]
		CYP3A4 and CYP2D6 enzymes	2.63 mg/mL (IC_50_)	Inhibited the gene expression of CYP3A4 and CYP2D6 enzymes	
Antioxidant	Aqueous	FMLP-stimulated neutrophils	10 µg/mL	Decreased luminol and lucigenin-enhanced chemiluminescence response	[42]
		Oxidant-scavenging in cell-free systems	10 and 0.62 µg/mL	Inhibited superoxide-induced chemiluminescence at 10 µg/mL and horseradish peroxidase/hydrogen peroxide-induced chemiluminescence at 0.62 µg/mL	
	L-canavanine, D-Pinitol	RAW 264.7 cells	0.5 mM (L-c), 10 mM (D-p)	Inhibited LPS-induced NO secretion without reducing cell numbers	[9]
	70% ethanol	Primary rat cortical neurons	0.1–7.5 µg/mL	Inhibited NMDA-induced ROS production without altering viability	[43]
	Aqueous	CHO, HepaRG, and A549	500 µg/mL	Protected cells from t-BHP-induced oxidative stress by scavenging ROS, preserving GSH/GSSG levels	[44]
			1 mg/mL	Potent scavenger of hydroxyl, superoxide, and hydrogen peroxide radicals	
Anti-inflammatory	70% ethanol	BV-2 and HAPI microglial cells	0.1–80 µg/mL	Inhibited IFN-γ-induced p-ERK1/2 and p-STAT-1α expression as well as NO and filopodia production (BV-2) Inhibited LPS+ IFN-γ induced ROS and NO production as well as iNOS expression (BV-2 and HAPI)	[43]
	Ethanolic	RAW 264.7 cells	200 µg/mL	Reduced NO, iNOS, IL-6, and TNF-α production; inhibited ERK1/2, STAT1-α, and NF-κB activation	[45]
	Sutherlandioside B-enriched	RAW 264.7 cells	200 µg/mL	Reduced ROS induced by LPS and IFN-γ	[45]
	Aqueous (leaves)	NRK-52E cells	0.4 mg/mL	Partially reduced TNF-α induced chemokine CCL5 expression	[46]
Antidiabetic	Dichloromethane (leaves)	α- and β-glucosidase enzymes	0.2 mg/mL	Significantly inhibited α- and β-glucosidase enzymes	[47]
	Aqueous	Hepatocytes	12.5 µg/mL	Prevented insulin resistance	[48]
Neuroprotection	Aqueous	MPP^+^-induced toxicity (SH-SY5Y cells)	20 µg/mL	Reduced ROS production	[49]
Immune Modulatory	Aqueous and ethanolic (leaves)	RAW 264.7 cells	200 µg/mL	Inhibited LPS-induced ERK1/2 and p38 phosphorylation; reduced NO, ROS, TNF-α, IL-6, GM-CSF, and G-CSF	[50]
			100 µg/mL	Reduced LPS-stimulated NO and ROS production	
			50 µg/mL	Reduced LPS-induced production of IL-1α	
			100, 150 and 200 µg/mL	Inhibited NF-κB activation by attenuating NF-κB p65 subunit phosphorylation on the Ser 536 residue	
	Ethanol		150 and 100 µg/mL	Reduced CD86 expression and inhibited COX-2	
			100 µg/mL	increased the CD206 cell surface marker expression	
	Polysaccharide-enriched fraction		200 µg/mL	Activated macrophages via TLR4 receptors and NF-κB signalling pathway	[51]
Anti-HIV	Aqueous (leaves)	HIV-1 RT enzyme	0.2 mg/mL	Inhibited HIV-1 reverse transcriptase enzyme by ≥50%	[47]
	Methanolic and aqueous	Human liver microsomes	10 mg/mL	Inhibited the metabolism of atazanavir	[52]
	Aqueous	CaCo-2 cells	10 mg/mL	Decreased atazanavir accumulation, bioavailability, and absorption	
	The triterpenoid glycoside-enriched fraction	CaCo-2 cells	500 µg/mL	Increased atazanavir accumulation and enhanced its bioavailability and absorption	
		Human liver microsomes		Decreased the atazanavir present (*p* < 0.001) in human liver microsomes	
	40% aqueous methanol	P450 enzymes	IC_50_ ranging from 17–160 μg/mL	Inhibited CYP1A2, CYP2A6, CYP2B6, CYP2C8, CYP2C9, CYP2C19, CYP3A4/5, and CYP3A4/5 with IC_50_ values of 41, 160, 20, 22.4, 23, 35.9, 17.5, and 28.3 μg/mL, respectively	[53]
		Hepatocytes	100 µg/mL	Reduced midazolam clearance by 40% by delaying the production of midazolam metabolites	
		LLC-PK1 cells stably transfected with human P-gp	324.8 µg/mL (IC_50_)	Inhibit P-gp	
		Human embryonic kidney 293 cells stably transfected with human OATP1B1 or OATP1B3	IC_50_	Inhibit OATP1B1 (10.4 µg/mL) and OATP1B3 (6.6 µg/mL)	
Cytotoxic	70% ethanol	MCF-7 and MCF-12A	10 mg/mL	Reduced cell growth and induced apoptosis in MCF-7 and MCF-12A	[54]
	Aqueous	MDBK and LLC-PK1 cells	6 mg/mL	Disrupted mitochondrial integrity, promoted apoptosis	[55]
		CHO and cervical neoplastic cells	3.5 mg/mL	Activated apoptosis	[56]
	70% ethanol and aqueous (tablet)	Normal T-lymphocytes	2.5 mg/mL	Induced necrosis, depleted ATP, inhibited caspase 3/7 activity, and caused DNA fragmentation	[57]
Antimutagenic	Ethyl acetate	TA97a, TA98, TA100, and TA102 strains	5, 10, 20% (*w*/*w*)	Exhibited antimutagenic effect against multiple strains	[58]
	L-arginine, GABA, D-Pinitol	TA97a, TA98, TA100, and TA102 strains	0.05–0.49 M	Exhibited antimutagenic activity against all four strains	
Pro-mutagenic	Methanol	TA98 and TA100 strains	10, 25, 50% (*w*/*w*)	Showed pro-mutagenic potential in TA98 with 2-acetamidofluorene and TA100 with aflatoxin B1	
Anti-tuberculosis	Dichloromethane–methanol (1:1)	Mycobacterium tuberculosis	IC_50_: 0.1–5.1 μg/mL	Inhibited the shikimate kinase enzyme	[11]
	α-Linolenic acid	Mycobacterium tuberculosis	3.7 µg/mL	Inhibited the shikimate kinase enzyme	
	Different DCM: MeOH fractions	Mycobacterium tuberculosis	0.3–94.3 µg/mL	Inhibited the shikimate kinase enzyme at varying IC_50_ values	
Anti-stress	Methanol, chloroform, and aqueous	Ovine adrenal mitochondria and microsomes (0.78 mM P450 in binding studies and 0.33 mM P450 in conversion)	2.4 (50 µL of 48 mg/mL) and 4.1 mg (50 µL of 82 mg/mL)	Decreased the binding of DOC, PROG, and PREG, as well as the conversion of PROG and PREG (methanol and chloroform, 4.1 mg) Inhibited substrate binding to CYP21 and CYP11B1 (aqueous, 2.4 mg)	[26]
	Triterpenoid fraction	P450 enzymes CYP17 and CYP21 enzymes	0.6 and 1.5 mg/mL	Inhibited the binding of PROG (0.6 mg/mL) and PREG (1.5 mg/mL)	[59]
	Methanol and aqueous		2.4 and 4.1 mg/mL	Methanol (4.1 mg/mL) and aqueous (2.4 mg/mL) extracts inhibited the binding of PROG	
	Aqueous	Adrenocortical microsomes	2.4 mg/mL	Inhibited PROG metabolism and the formation of DOC, 17-OH-PROG and deoxycortisol (46%)	
		COS-1 cells		Inhibited PREG and PROG metabolism and the formation of the hydroxysteroid intermediates than DHEA and A4	
	Methanol	COS-1 cells	2.6 mg/mL	Inhibited PROG binding to CYP17A1 and CYP21A2	[12]
		Human H295R adrenal cells	1 mg/mL	Decreased total steroid production under basal steroidogenesis and forskolin-stimulated steroidogenesis conditions Inhibited CYP17A1 and CYP11B1 and significantly reduced PROG, DOC, CORT, 17OH-PREG, 16OH-PROG, 11-DHC, 11OHA4, and A4 levels, while increasing DHEAS	
		COS-1 cells	0.5–0.75 mg/mL	Antagonised the effects of ALDO via the MR	
				Significantly repressed the IL-6 promoter after stimulation with PMA (10 ng/mL)	
	Sutherlandioside B	COS-1 cells	0.5 and 0.75 mg/mL	Suppressed NF-κB-driven gene expression while antagonising the effects of ALDO via the MR	[12]
		COS-1 cells	10 and 30 µM	Inhibited CYP17A1 activity toward PREG and PROG, as well as 3β-HSD2 activity toward PROG, and acted as selective glucocorticoid receptor agonists	
		In H295R cells	30 µM	Decreased CORT, A4, 11OH-A4, and 16OH-PROG, while increasing 11-DHC	
Antibacterial	Hexane	*Enterococcus faecali*, *Escherichia coli*, and *Staphylococcus aureus*	IC_50_ varying from 0.31 to 2.5 mg/mL	Inhibited bacteria with IC_50_ of 2.50 (Ef), 1.25 (Ec), and 0.31(Sa) mg/mL	[60]

**Table 3:** Ethnopharmacological and pharmacological properties of *L. frutescens* extracts and compounds in in vivo studies.

Pharmacological Activity	Extract/Compound	Experimental Models	Tested Concentrations	Experimental Outcomes	References
Anti-analgesic	Aqueous (shoot)	Hot-plate and acetic acid test models of pain in mice	50–800 mg/Kg	Produced analgesic effects against thermally and chemically induced nociceptive pain stimuli in mice	[3]
Anti-inflammatory		Fresh egg albumin-induced pedal oedema	50–800 mg/Kg	Inhibited fresh egg albumin-induced acute inflammation	[3]
Antidiabetic	Aqueous (shoot)	Streptozotocin-induced hyperglycemia in mice	50–800 mg/Kg	Inhibited streptozotocin-induced hyperglycemia	[3]
	Aqueous	Wistar rats fed a diabetogenic diet	0.01 mL/g rat weight	Increased glucose uptake into muscle and adipose tissue while significantly decreasing intestinal glucose uptake	[61]
Anti-convulsion	Aqueous (shoot)	Streptozotocin (PTZ)-induced seizures in mice	50–800 mg/kg	Protected the mice against PTZ-induced seizures	[27]
		Picrotoxin (PCT)-induced seizures in mice	50–800 mg/kg	Protected the mice against PCT-induced seizures	[27]
Anti-HIV	70% Ethanol	Rat hepatic and intestinal tissues	12 mg/kg	Increased intestinal and hepatic rat CYP3A2 expression levels after 5 days of exposure	[62]
		Rats	6 mg/kg	Altered the pharmacokinetics of nevirapine after 5 days of chronic exposure by reducing the AUC0–inf and Cmax	
Antioxidant/Spermatotoxic	Methanol	Wistar rats	2 mg/mL	Rapid progressive motility decreased, while slow-moving spermatozoa, catalase activity, and SOD activity significantly increased. The rise in SOD activity was associated with a reduction in MDA levels	[63]
Anti-nephrotoxicity	D-pinitol	Cisplatin-induced nephrotoxicity in mice	10–40 mg/kg/day	Prevented alterations in renal biomarkers, urine creatinine, serum blood urea nitrogen, and NO levels; improved histopathological alterations by preventing severe necrosis	[64]
		Cisplatin-induced nephrotoxicity in mice	10, 20, and 40 mg/kg/day	Altered cisplatin-induced changes in inflammatory markers by decreasing the levels of TNF-α, IL-1β, IL-6, and NO	

**Table 4:** Pharmacological properties of *L. frutescens* extracts in clinical trial studies.

Pharmacological Activity	Extracts	Experimental Model	Tested Concentrations	Experimental Outcomes	References
Anticancer	Aqueous	16 cancer patients (11 men and 5 women)	600 mg/day	Decreased fatigue in cancer patients (oral consumption).	[87]
Non-cytotoxic	leaf powder (capsules)	25 healthy adults	800 mg/day	Improved appetite in the treatment group. Overall, healthy adults tolerated 800 mg/d of Sutherlandia leaf powder well for three months.	[5]
Anti-HIV	Leaf powder	107 participants	2400 mg/day (1200 mg twice daily)	did not alter the viral load, and the CD4 T-lymphocyte count remained the same in the two arms.	[89]
